# Placenta accreta spectrum – variations in clinical practice and maternal morbidity between the UK and France: a population‐based comparative study

**DOI:** 10.1111/1471-0528.17169

**Published:** 2022-04-29

**Authors:** Stephen J. McCall, Catherine Deneux‐Tharaux, Loïc Sentilhes, Rema Ramakrishnan, Sally L. Collins, Aurélien Seco, Jennifer J. Kurinczuk, Marian Knight, Gilles Kayem

**Affiliations:** ^1^ National Perinatal Epidemiology Unit, Nuffield Department of Population Health University of Oxford Oxford UK; ^2^ Université de Paris CRESS U1153, INSERM, Obstetrical, Perinatal and Pediatric Epidemiology (EPOPé) Research Team Paris France; ^3^ Center for Research on Population and Health, Faculty of Health Sciences American University of Beirut Beirut Lebanon; ^4^ Department of Obstetrics and Gynecology Bordeaux University Hospital Bordeaux France; ^5^ Nuffield Department of Women's and Reproductive Health University of Oxford Oxford UK; ^6^ Fetal Medicine Unit John Radcliffe Hospital Oxford UK; ^7^ Clinical Research Unit Paris‐Descartes Necker/Cochin Paris France; ^8^ Hôpital Trousseau Assistance Publique –Hôpitaux de Paris (APHP), Sorbonne Université Paris France

**Keywords:** conservative management, haemorrhage, hysterectomy, management, placenta accreta spectrum

## Abstract

**Objective:**

To compare the management and outcomes of women with placenta accreta spectrum (PAS) in France and the UK.

**Design:**

Two population‐based cohorts.

**Setting:**

All obstetrician‐led hospitals in the UK and maternity hospitals in eight French regions.

**Population:**

A cohort of 219 women with PAS in France and a cohort of 154 women with PAS in the UK.

**Methods:**

The management and outcomes of women with PAS were compared between the UK and France.

**Main outcome measures:**

Median blood loss, severe postpartum haemorrhage (≥3 l), postpartum infection and damage to surrounding organs.

**Results:**

The management of PAS differed between the two countries: a larger proportion of women with PAS in the UK had a caesarean hysterectomy compared with France (43% vs 26%, *p* < 0.001), whereas in France a larger proportion of women with PAS received a uterus‐preserving approach compared with the UK (36% vs 19%, *p* < 0.001). The total median blood loss in the UK was 3 l (IQR 1.7–6.5 l), compared with 1 l (IQR 0.5–2.5 l) in France; more women with PAS had a severe postpartum haemorrhage (PPH) in the UK compared with women with PAS in France (58% vs 21%, *p* < 0.001) [Correction added on 06 May 2022, after first online publication: ‘24 hour’ has been changed to ‘total’ in the preceding sentence]. There was no difference between the UK and French populations for postpartum infection or organ damage.

**Conclusions:**

The UK and France have very different approaches to managing PAS, with more women in France receiving a uterine‐conserving approach and more women in the UK undergoing caesarean hysterectomy. A life‐threatening haemorrhage was more common in the UK than in France, which may be the result of differential management and/or the organisation of the healthcare systems.

In women with placenta accreta spectrum, severe haemorrhage was more common in the UK than in France.

**Tweetable abstract:**

In women with placenta accreta spectrum, severe haemorrhage was more common in the UK than in France.

## Tweetable abstract

In women with placenta accreta spectrum, severe haemorrhage was more common in the UK than in France.

**Linked article**: This article is commented on by Amarnath Bhide, pp. 1686 in this issue. To view this minicommentary visit https://doi.org/10.1111/1471-0528.17170.

## 1 INTRODUCTION

Placenta accreta spectrum (PAS) includes placenta accreta, placenta increta and placenta percreta, and is traditionally characterised histologically by a total or partial absence of decidua and placental adherence to or invasion of the myometrium.^1^ PAS becomes a clinical problem after birth, when the placenta does not physiologically detach from the uterus and the forcible removal of the placenta is followed by massive obstetric haemorrhage (MOH), leading to further morbidity and risk of maternal death.^2^, ^3^, ^4^ PAS incidence has accelerated in recent decades,^5^, ^6^, ^7^ simultaneously with the rise in caesarean section rates, which have surpassed 30% in many high‐resource countries.^8^, ^9^, ^10^

Early detection and planned management have been associated with improved maternal outcomes. The antenatal detection of PAS is critical for the prevention of maternal morbidity arising from PAS,^11^, ^12^ as it enables appropriate management from a multidisciplinary team with experience in managing the complexity presented by PAS within a well‐equipped tertiary centre.^11^, ^12^, ^13^, ^14^ The management of PAS can be broadly subdivided into two main approaches: conservative approaches or caesarean hysterectomy. However, comparison of these approaches for women with PAS has not been robustly investigated.^15^, ^17^

Caesarean hysterectomy is considered the standard approach for managing PAS,^11^, ^18^ and forms the mainstay of the management of PAS for many authorities and clinicians internationally, including in the UK.^3^ Previous studies have shown caesarean hysterectomy to be a life‐saving treatment for women where other uterus‐conserving surgeries fail.^19^, ^20^ Others have used an approach to conserve the uterus where the placenta is left in situ, either partially or completely, without any attempted removal.^17^, ^21^ This approach is the most common method of conservative management.^22^ Given the rarity, potential severity and heterogeneity in management, a randomised controlled trial would be extremely difficult to perform in this area. Therefore, an international comparison between countries with different management policies of PAS may help to answer whether a conservative approach is effective and safe.

Currently, a uterine‐conserving approach is frequently used in women with PAS in France. French guidelines propose two options: treatment to conserve the uterus or caesarean hysterectomy.^23^ Conversely, the UK guidelines recommend caesarean hysterectomy as the standard management and leaving the placenta in situ only for women desiring uterine preservation or when the surgical team considers caesarean hysterectomy inappropriate.^24^ This cross‐country variation offers an opportunity to study and compare the outcomes from two countries. This study aimed to compare the management and maternal outcomes of women with PAS in France and in the UK.

## 2 METHODS

### 2.1 Study population

This was a binational population‐based secondary analysis study of PAS using data from two population‐based cohort studies from the UK and France.^25^, ^26^, ^27^, ^28^ The data were collected nationally in the UK and from eight regions within France. Data were prospectively collected using a national obstetric surveillance system in the UK (UK Obstetric Surveillance System, UKOSS) and a prospective cohort study in France (PACCRETA).

### 2.2 UKOSS (UK)

The UK data were collected using the UKOSS system using methods that have been previously described.^29^ In brief, women meeting the case definition were identified nationally in the UK during the period from May 2010 to April 2011. Women with PAS were identified using the case definition in Table 1. Anonymised data were collected using a paper data collection form and a nominated reporter in each maternity hospital in the UK completed this form using the woman’s hospital records. The UKOSS data have been published previously.^3^, ^25^

**TABLE 1:** Case definitions from the respective studies

UKOSS case definition	PACCRETA case definition
Women were included as having PAS if they met either of the following criteria: Placenta accreta/increta/percreta diagnosed histologically following hysterectomy or post‐mortem. or An abnormally adherent placenta requiring *active management*, including conservative approaches where the placenta is left in situ. *Excluded*: women who had a manual placental removal with minimal or moderate difficulty but required no additional active management. *Active management*: this is when some other manipulation is required to remove the placenta and the placenta can only be partially removed or is removed piecemeal, with clear documentation that the clinician did not feel it was fully removed.	Women were included as having PAS if they met any of the following criteria: Manual removal of the placenta, partially or totally, impossible and no cleavage plane between part or all of the placenta and the uterus. Massive bleeding from the implantation site after forced placental removal in the absence of another cause of postpartum haemorrhage (PPH). Histological confirmation of PAS on a hysterectomy specimen. Signs of PAS at laparotomy in women with suspected PAS on prenatal imaging.
**Harmonised definition for comparative study**	
Women were included as having abnormally invasive placenta if they met either of the following criteria: Placenta accreta/increta/percreta diagnosed histologically following hysterectomy or post‐mortem. or An abnormally adherent placenta requiring *active management*, including conservative approaches where the placenta is left in situ. *Excluded*: Women who had a difficult manual removal of the placenta but did not require *active management* were excluded from the study as there was no evidence to confirm PAS. *Active management*: same as described in the UKOSS case definition above.	

### 2.3 PACCRETA (France)

The methodology of the PACCRETA study has been described in the published protocol.^26^ In summary, this population‐based study identified women meeting the case definition (Table 1) during the period from November 2013 to October 2015, from 176 centres across eight regions of France, where there were 520 114 maternities, which represents 30% of the national total over the study period. Each centre had a nominated clinician that identified women who met the case definition. In addition, delivery suite logs and electronic records were checked to maximise case ascertainment. Data collected from the medical records of each woman were entered onto a web‐based data collection form.^27^

### 2.4 Case definitions

The case definitions for the individual studies conducted in France and the UK differed. A common case definition was devised to provide a harmonised data set of women with PAS, which involved selecting women in France that met the stricter UKOSS definition (Table 1).

#### 2.4.1 Specific classification for variables

Information on the comparability of the data set is available in the supporting information. The grading of PAS (accreta, increta and percreta) was classified into two categories: a category of placenta percreta and a category containing both placenta increta and placenta accreta, on the basis that placenta increta and placenta accreta are often largely indistinguishable clinically, and because some women with placenta left in situ had no histological uterine examination when a hysterectomy was avoided. Any history of uterine surgery was combined into a variable that included previous caesarean section, myomectomy, cavity breach, dilation and curettage, previous surgical termination of pregnancy and evacuation of retained products of conception. Uterotonics for treatment of haemorrhage included oxytocin, ergometrine, misoprostol, sulprostone and other synthetic prostaglandins.

#### 2.4.2 Management, and maternal and infant outcomes

Conservative management was defined as placenta left in situ, either completely or partially, in women who did not have a caesarean hysterectomy. Surgical management for haemorrhage included hysterectomy, pelvic arterial embolisation, uterine balloon tamponade and other conservative surgeries, including uterine compression sutures and arterial ligation. Hysterectomy was categorised into total hysterectomy (occurring at any point), caesarean hysterectomy (occurring after a caesarean section, within 4 h of birth for women in the UK and verified as a caesarean hysterectomy from the operative report for French women) and hysterectomy after placenta left in situ (conservative approach). Planned hysterectomy was indicated in the medical notes if hysterectomy had been planned before delivery. Time of hysterectomy was also available in both data sets.

The outcomes were: median estimated total blood loss; severe postpartum haemorrhage (≥3000 ml); major postpartum haemorrhage (≥2000 ml); red blood cell (RBC) transfusion; massive blood transfusion (≥6 units of RBC); damage to the bowel, urinary tract or bladder; postpartum infection; Intensive Therapy Unit (ITU) admission; and maternal death.^30^ Damage to the urinary tract, bowel or bladder was combined into one category. Postpartum infection and damage to the surrounding organs were extracted from free‐text responses to the question of ‘did woman have any other morbidity?’ in the UK, and the French data were based on specific questions. The primary infant outcome was perinatal death.

#### 2.4.3 Missing data

Women in France who did not have a postpartum haemorrhage (PPH) did not have any blood loss value entered; the estimated blood loss for French women without a PPH was imputed to be 500 ml, as above this threshold a PPH would have been recorded in the data collection form.^23^ In addition, this approach avoided possible bias towards French management.

### 2.5 Statistical analysis

The incidence of PAS was calculated per 10 000 maternities, according to the estimated number of maternities in the UK and the reported number of maternities from each maternity unit in France, that occurred during the study period. The confidence intervals were estimated using the exact binomial distribution.

A comparative analysis was undertaken in women who had PAS in both France and the UK. The women’s characteristics, medical history, obstetric and haematological management and perinatal and maternal outcomes were compared between France and the UK. Normality was assessed using histograms. Normally distributed continuous variables were presented as means with standard deviations, and skewed continuous variables were presented as medians with interquartile ranges. The following statistical tests were used where appropriate: Student’s *t*‐test, Wilcoxon rank‐sum test, Kruskal–Wallis test and chi‐square test or Fisher’s exact test. A *p*‐value of <0.05 was used to determine statistical significance.

Sensitivity analyses were undertaken to assess results in subpopulations, which included women who had placenta percreta, women with PAS suspected antenatally and women with placenta praevia detected antenatally. All statistical analyses were conducted using STATA v15.1 (Stata Corp. LLC, College Station, TX, USA).

#### 2.5.1 Patient and public involvement

The patients and public were involved in the design and interpretation of the UKOSS placenta accreta study as part of the UKOSS Steering Committee. There was no patient and public involvement in the PACCRETA study.

## 3 RESULTS

**FIGURE 1:**
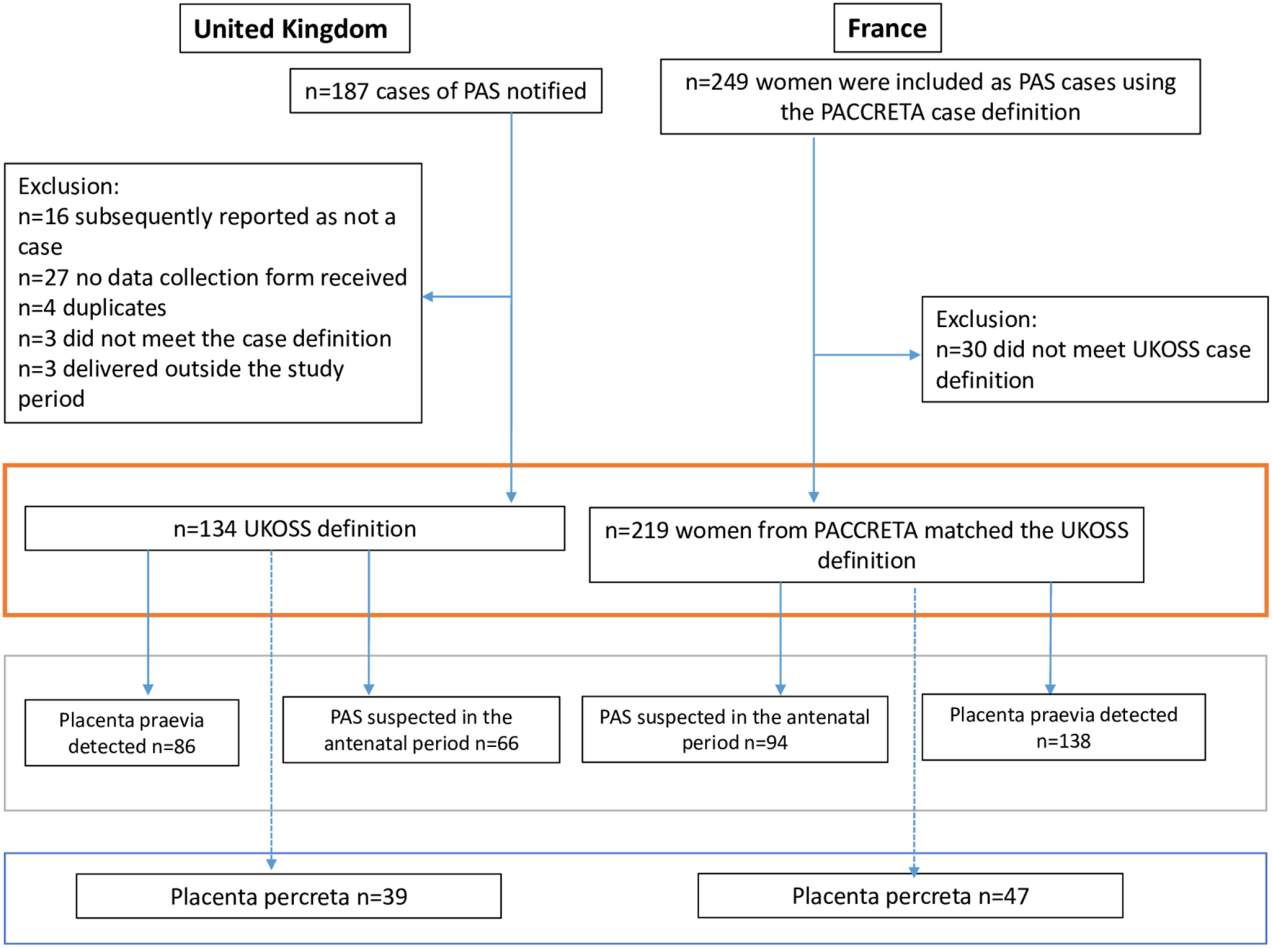
Study selection

The derived study population in the UK and France is shown in Figure 1. In the UK, there were 134 women with PAS who met the case definition during the period from May 2010 to April 2011, among 798 634 maternities. This gave an estimated incidence of PAS in 1.7 women per 10 000 maternities (95% CI 1.4–2.0). After the harmonisation of definitions, 219 women in the PACCRETA study met the same case definition over a 2‐year period in 2013–2015, among 520 114 maternities. This gave an estimated incidence of PAS in 4.2 women per 10 000 maternities (95% CI 3.7–4.8); there was a statistically significant difference between the UK and France (*p* < 0.001).

### 3.1 Characteristics of women with PAS

The mean age at delivery, the proportion of women who were obese and the proportion of women who smoked during pregnancy between the PAS cohorts in the UK and France did not statistically differ (Table 2). In women who had PAS and had a previous pregnancy, a statistically significantly higher proportion of women in the UK had at least one previous caesarean section compared with women in France (93% vs 80%, *p* = 0.003), whereas a higher proportion of women in France had other previous uterine surgery compared with women in the UK (44% vs 29%, *p* = 0.007).

**TABLE 2:** Characteristics of women with PAS in the UK and France

	UK *n* (%)	France *n* (%)	*p*
	*N* = 134	*N* = 219	
Age (years), mean (SD)	34. 6 (5.6)	34.5 (5.1)	0.833
BMI (kg/m^2^)			
<25	60 (45.8)	119 (55.3)	0.111
25 to <30	42 (32.1)	48 (22.3)	
≥30	29 (22.1)	48 (22.3)	
Missing	3	4	
Smoking status			
Did not smoke during pregnancy	107 (80.5)	165 (78.6)	0.675
Smoked during pregnancy	26 (19.5)	45 (21.4)	
Missing	1	9	
Country of birth			
Not France	–	87 (42.4)	
France	–	118 (57.6)	
Missing	–	14	
Ethnicity			
White	99 (74.4)	–	
Non‐white	34 (26.6)	–	
Missing	1	–	
Parity			
0	12 (9)	37 (16.9)	0.082
1	39 (29.1)	66 (30.1)	
2+	83 (61.9)	116 (53.0)	
History of PPH			
No	111 (91)	145 (79.7)	0.008
Yes	11 (9)	37 (20.3)	
N/A (nulliparous)	12	37	
Previous caesarean section			
0	9 (7.4)	36 (19.8)	0.011
1	63 (51.6)	80 (44)	
2+	50 (41.0)	66 (36.3)	
N/A (nulliparous)	12	37	
Previous uterine surgery^a^			
No	94 (70.7)	123 (56.2)	0.007
Yes	39 (29.3)	96 (43.8)	
Missing	1	0	
Previous uterine surgery and caesarean section			
Yes	129 (96.3)	207 (94.5)	0.457
Hypertensive disorder during pregnancy			
Yes	6 (4.6)	20 (9.1)	0.116
Missing	3	0	
Placenta praevia detected prior to birth			
Yes	86 (64.7)	138 (63.0)	0.755
Missing	1	0	
Multiple pregnancy			
Yes	4 (3.0)	10 (4.6)	0.460
PAS suspected prior to birth			
Yes	66 (49.6)	94 (44.8)	0.379
Missing	1	9	
PAS type			
Placenta accreta/increta	94 (70.7)	172 (78.5)	0.096
Placenta percreta	39 (29.3)	47 (21.5)	
Missing	1	0	

Abbreviations: BMI, body mass index; PAS, placenta accreta spectrum; PPH, postpartum haemorrhage.

^a^Includes: myomectomy; cavity breached; dilation and curettage; previous surgical termination of pregnancy; and evacuation of retained products of conception (excluding caesarean section). Descriptive statistics calculated excluding missing data.

In both the UK and France, approximately half of the women had PAS suspected prior to delivery. There was no statistically significant difference in the grade of PAS between women in France and women in the UK: 29% and 22% had a final diagnosis of placenta percreta in the UK and France, respectively.

### 3.2 PAS management

In the UK, over three‐quarters of women (76%) had an attempted manual removal of the placenta, whereas this occurred for 68% of women in France (*p* < 0.001). Women with PAS in the UK were more frequently managed with caesarean hysterectomy than women in France (43% vs 26%, *p* < 0.001), whereas a lower proportion of women had their placenta left in situ in the UK compared with France (19% vs 36%, *p* < 0.001) (Table 3). In women that had placenta left in situ, approximately one‐third of women in the UK and one‐fifth of women in France went on to have a hysterectomy.

**TABLE 3:** Mode of birth and management of women with PAS in the UK and France

Management	UK *n* (%)		France *n* (%)	*p*
	*N* = 134		*N* = 219	
Caesarean section				
Yes	118 (88.1)		187 (85.4)	0.477
Uterotonics used as treatment or prophylaxis^a^				
Used	109 (81.3)		164 (75.2)	0.182
Missing	0		1	
Attempt to manually remove the placenta				
Attempt	102 (76.1)		149 (68.3)	<0.001
No attempt	32 (23.9)		69 (31.7)	
Missing	0		1	
In women with antenatal care suspicion of PAS: attempt to manually remove the placenta				
Attempt	39 (59.1)		26 (28.0)	<0.001
No attempt	27 (40.9)		67 (72.0)	
Missing	0		1	
Caesarean hysterectomy^b^				
Yes	58 (43.3)		57 (26.0)	<0.001
No	76 (56.7)		162 (74.0)	
Total hysterectomy				
Yes	79 (59.0)		83 (38.1)	<0.001
No	55 (41.0)		135 (61.9)	
Missing	0		1	
Hysterectomy planned				
Yes	38 (48.1)		9 (10.8)	<0.001
No	41 (51.9)		74 (89.2)	
Time between birth and hysterectomy				
≤48 h	73 (92.4)		70 (84.3)	0.111
>48 h	6 (7.6)		13 (15.7)	
Conservative approach: placenta left in situ^c^				
Yes	26 (19.4)		79 (36.1)	<0.001
No	108 (80.6)		140 (63.9)	
How much placenta left in situ				
Complete	18 (69.2)		40 (50.6)	0.098
Partial	8 (30.8)		39 (49.4)	
Hysterectomy after placenta left in situ				
Yes	8 (30.8)		18 (22.8)	0.413
Time between birth and hysterectomy				
≤48 h	3 (37.5)		7 (38.9)	0.946
>48 h	5 (62.5)		11 (61.1)	
Methotrexate used				
Yes	7 (26.9)		0 (0.0)	<0.001
Missing	0		1	

^a^ Use of misoprostol, ergometrine, syntocinon, sulprostone or other prostaglandin.

^b^ Caesarean hysterectomy was defined as women who had a hysterectomy within 4 h of caesarean section in the UK; for the French data, caesarean hysterectomy was indicated in the operative record.

^c^ Conservative approach was verified from the medical notes in France; for the UK data this was derived as women who had placenta left in situ without caesarean hysterectomy. Descriptive statistics calculated excluding missing data.

### 3.3 The maternal and infant outcomes of women with PAS

The maternal and infant outcomes are shown in Table 4. The median estimated total blood loss for women with PAS was 3050 ml (IQR 1700–6500 ml) in the UK, whereas it was lower in France, with a median value of 1000 ml (IQR 500–2500 ml; *p* < 0.001). Over half of the women with PAS in the UK had a severe PPH of ≥3000 ml, whereas only a fifth of women in France experienced this level of haemorrhage (*p* < 0.001). Among women who had a hysterectomy after an attempted conservative approach, the median blood loss was 2000 ml (IQR 500–4000 ml). The difference in blood loss between the UK and France remained consistent when the analysis was restricted to women with placenta percreta, women with PAS suspected antenatally and women with placenta praevia detected antenatally (Tables S1–S5).

**TABLE 4:** Maternal and infant outcomes in women with PAS in the UK and France

Maternal outcomes		UK *n* (%)	France *n* (%)	*p*
		*N* = 134	*N* = 219	
Total blood loss (ml)	Median (IQR)	3050 (1700–6500)	1000 (500–2500)	<0.001
Severe postpartum haemorrhage (ml)	<3000	57 (42.5)	165 (79.3)	<0.001
	≥3000	77 (57.5)	43 (20.6)	
	Missing	0	11	
Major postpartum haemorrhage (ml)	<2000	37 (27.6)	143 (68.8)	<0.001
	≥2000	97 (72.4)	65 (31.1)	
	Missing	0	11	
Whole blood or RBC received, *n* (%)		102 (76.1)	111 (50.7)	<0.001
In women who received whole blood or RBC	Median (IQR) unit	7 (4–12)	5 (3–10)	0.135
Massive blood transfusion (units)	≥6	65 (63.7)	52 (48.6)	0.028
	<6	37 (36.3)	55 (51.4)	
	Missing	0	4	
Pelvic arterial embolisation	Used	33 (24.6)	49 (22.4)	0.627
Other conservative surgery for haemorrhage^a^	Used	28 (20.9)	31 (14.2)	0.100
Uterine balloon tamponade	Used	33 (24.6)	33 (15.1)	0.025
Postpartum infection^b^	Yes	3 (2.2)	3 (1.4)	0.332
	Missing	0	3	
Damage to bowel, urinary tract or bladder	Yes	10 (7.5)	17 (7.9)	0.889
	Missing	0	3	
ITU admission	Yes	92 (68.7)	65 (30)	<0.001
	Missing	0	2	
Maternal mortality	Yes	0 (100)	1 (0.5)^c^	0.999

Infant outcomes^d^		UK *n*(%)	France *n* (%)	
		*N* = 138	*N* = 229	
Perinatal mortality	No	132 (98.5)	222 (98.7)	0.759
	Yes	2 (2.0)	3 (1.3)	
	Missing	4	4	

^a^ Includes: arterial ligation and uterine compression sutures.

^b^ UK data were extracted from free‐text response to ‘did woman have any other morbidity?’ and French data were based from specific questions on infective symptoms, haematological culture, and symptoms of fever and septic shock.

^c^ The cause of death was haemorrhagic shock, and the secondary factors that led to death were the attempted manual removal of the placenta and a failed embolisation.

^d^ The denominator is the number of infants. Descriptive statistics calculated excluding missing data.

Approximately three‐quarters of women with PAS received an RBC transfusion in the UK, whereas half of women received an RBC transfusion in France (*p* < 0.001). Nearly two‐thirds of women in the UK with PAS had a massive blood transfusion, compared with half of the women with PAS in France (64% vs 49%, *p* = 0.028). Further information on haematological management is available in Table S6.

In both countries, approximately 20% of women with PAS underwent pelvic arterial embolisation. The UK had a statistically significantly higher proportion of women managed with a uterine balloon tamponade, compared with France (25% vs 15%, *p* = 0.025, respectively). There were similar proportions of women with PAS who had damage to their bowels, urinary tract or bladder. There was no difference in the proportion of women with an infection between the UK and France (2% vs 1%, *p* = 0.332). ITU admission for women with PAS was higher in UK than for women in France (69% vs 30%, *p* < 0.001). One woman died, from haemorrhagic shock, which was caused by an attempted manual removal of the placenta and a failed embolisation. In women who had PAS, there was no significant difference in the perinatal mortality between the UK and France.

## 4 DISCUSSION

This binational study has shown that the management and maternal outcomes were different between the two cohorts of women with PAS in the UK and France, despite having similar proportions of placenta percreta and antenatally suspected cases of PAS. In particular, the majority of women with PAS were managed with planned hysterectomy in the UK, while in France, a left in situ approach to conserve the uterus was more commonly attempted. The UK had a larger proportion of women who had a severe PPH. The difference in severe PPH between the UK and France remained when the analysis was restricted to women with PAS suspected antenatally, women with su placenta praevia and women with placenta percreta.

### 4.1 Interpretation

Similar to previous findings, this study showed that the primary management for PAS in the UK was peripartum hysterectomy.^3^, ^24^ A smaller and older case series showed that the conservative management of PAS in France was used in 25% of women affected,^17^ whereas the findings from this study showed that this approach is now used for a third of the women with PAS in France.

It is surprising that an attempted manual removal of the placenta occurred in women with an antenatal suspicion of PAS. Nevertheless, this occurred in a large proportion of women in both countries. These findings suggest that clinicians in the UK and France did not adhere to the clinical guidelines of not attempting a manual removal of the placenta and leaving the placenta undisturbed when PAS is suspected.^16^, ^31^, ^32^ These data indicate that the implementation of guidelines requires to be strengthened and women should be referred to tertiary centres with multidisciplinary teams experienced in PAS if there is suspicion of PAS.^32^

Previous studies have shown that women had preserved future fertility and reduced haemorrhage risk when PAS was managed conservatively.^15^, ^21^ However, these studies were not population‐based and lacked an appropriate comparison group.^17^, ^33^, ^34^ The greater use of a conservative approach in France may be a potential explanation for the lower blood loss observed in women with PAS compared with the UK.

Assuming that the complexity of surgery is the same for women in France and the UK, the lack of centralisation of care for PAS in the UK may be a partial explanation for the higher proportion of women with severe PPH, where the UK primarily manages PAS with caesarean hysterectomy. In women with PAS, hysterectomies were performed in the majority of centres in the UK at the time of data collection for this study. A recent study in the UK found that one‐third of maternity centres in the last 5 years managed fewer than one case of PAS per year.^35^ In contrast, PAS care was centralised into specialist centres in France; thus, the clinical teams in France were more experienced in managing women with PAS than the clinical teams in the UK. Ruiz and Chen^36^ have shown that there were higher complication, transfusion and mortality rates for hysterectomies performed by inexperienced surgeons compared with experienced surgeons.^36^ Furthermore, maternal outcomes improve within the same centre as clinical teams become more experienced in managing PAS.^37^ Colleagues have shown that women who had modified radical peripartum caesarean hysterectomy conducted by highly skilled and experienced surgical teams had better outcomes compared with normal surgical approaches; accordingly, the experience and skill set of the surgical team matters.^38^ In addition, obstetricians and radiologists perform the majority of antenatal imaging in France, whereas a midwife or sonographer performs these scans in the UK. This may have led to varying levels of confidence in the PAS diagnosis between France and the UK, where the manual attempted removal of the placenta was performed more readily in the UK compared with France. Thus, a potential explanation for the difference in blood loss may be the result of differences in the healthcare system. Although planned, the UK health system has yet to centralise PAS management into multidisciplinary teams that regularly perform complex surgeries for PAS. The findings of this study recommend the immediate implementation of these plans in the UK. Other countries should also consider centralising PAS care.

Conversely, the lower rates of haemorrhage in France in women who had a conservative approach has biological plausibility. After delivery, the blood flow to the uterus decreases, which will result in necrosis of the placenta and either expulsion or reabsorption of the placenta. Without a rupture, failed conservative management or trauma to the uterus, there is less likely to be a spontaneous haemorrhage,^18^ whereas even a planned hysterectomy for placenta percreta is likely to result in major blood loss. Yet, a conservative approach is only possible where postpartum follow‐up is feasible, as one‐fifth of women in France with conservative management had a delayed hysterectomy, and these women had severe blood loss. Future studies are required for comparison between those with a planned hysterectomy and those with planned conservative management.

### 4.2 Limitations and strengths

Both the UK and French studies had different case definitions, so even with considerable effort to harmonise the definitions of the two studies it was possible that the populations slightly differed. It may be that the UK study comprised more severe cases of PAS compared with the French study, which could be a potential explanation for the differences in incidence. Future studies should adopt the same definition or be designed together in a single prospective study, to allow more straightforward comparison. However, despite this limitation, when the analysis was restricted to women with placenta percreta there was still a difference in the estimated blood loss between the two countries. Studies have highlighted issues with subclassifying PAS; as a result, future prospective studies should use the International Federation of Gynaecology and Obstetrics (FIGO) guidelines and current evidence to allow for accurate subclassification, harmonisation and comparability.^39^, ^40^

A randomised controlled trial is the most robust method to examine the causal effect of management on outcomes, but this would be difficult to conduct in this clinical scenario. In addition, future studies could be further strengthened by including women’s satisfaction with care as an outcome or other patient‐centred outcomes.

Another limitation to note is the management of PAS has changed since these data were collected.^32^ Although the UK and French studies were conducted during a comparable time period, allowing appropriate comparison, the evolution of knowledge, awareness and management of PAS across this time period may be a partial explanation for the differences in outcomes between the countries.^41^ It should be noted that the data from these two studies may not reflect current practice, yet these data illustrate that differences in health system‐affected outcomes for women with PAS.

## 5 CONCLUSION

This binational study showed a substantial difference in blood loss between France and the UK. This may be the result of differences in clinical practice or the structure of the health care system. Importantly, the centralisation of care into specialist centres with skilled multidisciplinary teams is required to optimise the outcomes for women with PAS. Uterine‐preserving management may have resulted in a greater number of women retaining their potential fertility and reduced the likelihood of a life‐threatening haemorrhagic event. However, if the conservative management approach fails, it is likely to result in severe maternal morbidity, which reinforces the recommendations for the regular close monitoring of these women.

## DISCLOSURE OF INTERESTS

None declared. Completed disclosure of interests form available to view online as supporting information.

## CONTRIBUTION TO AUTHORSHIP

GK, CDT, MK and SM contributed to the conceptualisation and investigation. GK was the scientific lead for the PACCRETA study. MK was the scientific lead for the UKOSS study. GK and MK were the data curators. SM completed the primary analysis and wrote the first draft. AS and RR validated the data analysis. GK, CDT, MK, SM, SC, JK and LS provided methodological input into the study. All authors reviewed and edited the article.

## DETAILS OF ETHICS APPROVAL

PACCRETA study: The Committee for the Protection of Patients (AOR12156), the Consultative Committee on the Treatment of Personal Health Data for Research Purposes and the National Data Protection Authority (CNIL no. DR‐2013‐427) approved the study protocol. UKOSS: the UKOSS methodology has been approved by the London Multi‐Centre Research Ethics Committee (MREC) (MREC ref. 04/MRE02/45).

## Supporting information


Supplementary Tables (S1‐S6)

Appendix S1

Data S1

Data S2

Data S3

Data S4

Data S5

Data S6

Data S7

Data S8

Data S9

## Data Availability

Request for access to the data should be directed to the steering committee of the relevant country. Requests for access to the French data (PACCRETA) should be directed to epope@inserm.fr. Requests for access to the UK dataset will be considered by the National Perinatal Epidemiology Unit Data Sharing committee. Access to the UK data can be requested from general@npeu.ox.ac.uk.^a^
