# Supplementary material for: Placenta accreta spectrum – variations in clinical practice and maternal morbidity between the UK and France: a population‐based comparative study
Source: BJOG. 2022 Apr 29;129(10):1676–85. doi: 10.1111/1471-0528.17169 (PMC9544707; doi:10.1111/1471-0528.17169)
Supplement: Supplementary file 1 — Supplementary Tables (S1‐S6) [file BJO-129-1676-s003.docx]

Table S1. Current pregnancy characteristics of women with PAS suspected in the antenatal period.

|  |  | UK n (%)  n=66 | | France n (%) n=94 | | P-value |
| --- | --- | --- | --- | --- | --- | --- |
| Age mean (SD) |  | 34.1 | (5.4) | 34.7 | (4.4) | 0.458 |
| BMI (kg/m^2^) | <25 | 30 | (46.2) | 42 | (46.2) | 0.169 |
|  | ≥25 & <30 | 23 | (35.4) | 22 | (24.2) |  |
|  | ≥30 | 12 | (18.5) | 27 | (29.7) |  |
|  | *Missing* | 1 |  | 3 |  |  |
| Smoking status | Did not smoke during pregnancy | 49 | (75.4) | 74 | (83.1) | 0.235 |
|  | Smoked during pregnancy | 16 | (24.6) | 15 | (16.9) |  |
|  | *Missing* | 1 |  | 5 |  |  |
| Country of birth | Not France | - | - | 46 | (52.9) |  |
|  | France | - | - | 41 | (47.1) |  |
|  | *Missing* | - | - | 7 | - |  |
| Ethnicity | White | 51 | (77.3) | - | - |  |
|  | Non-white | 15 | (22.7) | - | - |  |
|  | *Missing* | 0 |  | - |  |  |
| Parity | Zero | 1 | (1.5) | 4 | (4.3) | 0.634 |
|  | 1 | 16 | (24.2) | 25 | (26.6) |  |
|  | 2+ | 49 | (74.2) | 65 | (69.1) |  |
| *History of PPH* | No | 58 | (89.2) | 74 | (82.2) | 0.226 |
|  | Yes | 7 | (10.8) | 16 | (17.8) |  |
|  | N/A (nulliparous) | 1 |  | 4 |  |  |
| *Previous caesarean section* | Zero | 0 | (0) | 4 | (4.4) | 0.308 |
|  | 1 | 30 | (46.2) | 37 | (41.1) |  |
|  | 2+ | 35 | (53.8) | 49 | (54.4) |  |
|  | N/A (nulliparous) | 1 |  | 4 |  |  |
| *Previous uterine surgery* | No | 50 | (75.8) | 57 | (60.6) | 0.045 |
|  | Yes | 16 | (24.2) | 37 | (39.4) |  |
|  | *Missing* | 0 |  | 0 |  |  |
| *Previous uterine surgery and caesarean section* | Yes | 66 | (100) | 94 | (100) | - |
| Hypertensive disorder during pregnancy | Yes | 0 | (0.0) | 5 | (5.3) | 0.079 |
|  | *Missing* | 1 |  | 0 |  |  |
| Placenta praevia detected prior to delivery | Yes | 64 | (97.0) | 86 | (91.5) | 0.159 |
|  | *Missing* | 0 |  | 0 |  |  |
| Multiple pregnancy | Yes | 0 | (0.0) | 1 | (1.1) | 0.999 |
| PAS type | Placenta accreta/ increta | 42 | (64.6) | 53 | (56.4) | 0.298 |
|  | Placenta percreta | 23 | (35.4) | 41 | (43.6) |  |
|  | *Missing* | 1 |  | 0 |  |  |

Descriptive statistics calculated excluding the missing.

Table S2. Obstetric and haematological management of women with PAS suspected in the antenatal period.

|  |  | Abnormally invasive placenta suspected in antenatal period | | | | |
| --- | --- | --- | --- | --- | --- | --- |
| **Delivery and management** |  | UK n(%) n=66 | | France n(%) n=94 | | P-value |
| Termination of pregnancy | Yes | 0 | (0.0) | 1 | (1.1) | 0.999 |
| Gestational age at birth | <34 weeks | 12 | (18.2) | 19 | (20.2) | 0.044 |
|  | ≥34 & <37 weeks | 25 | (37.9) | 51 | (54.3) |  |
|  | ≥37 weeks | 29 | (43.9) | 24 | (25.5) |  |
| Planned caesarean section | Yes | 64 | (97.0) | 85 | (90.4) | 0.107 |
| Caesarean section | Yes | 65 | (98.5) | 94 | (100) | 0.231 |
| **Medical management** |  |  |  |  |  |  |
| Uterotonics used as treatment or prophylaxis* | Used | 52 | (78.8) | 59 | (63.4) | 0.038 |
|  | *Missing* | 0 |  | 1 |  |  |
| Attempt to manually remove the placenta | *Attempt* | 39 | (59.1) | 26 | (28.0) | <0.001 |
|  | *No attempt* | 27 | (40.9) | 67 | (72.0) |  |
|  | *Missing* | 0 |  | 1 |  |  |
| Caesarean Hysterectomy | Yes | 34 | (51.5) | 28 | (29.8) | 0.005 |
|  | No | 32 | (48.5) | 66 | (70.2) |  |
| Total Hysterectomy | Yes | 43 | (65.2) | 45 | (47.9) | 0.031 |
|  | No | 23 | (34.8) | 49 | (52.1) |  |
| *Hysterectomy planned* | Yes | 32 | (74.4) | 7 | (15.6) | <0.001 |
|  | No | 11 | (25.6) | 38 | (84.4) |  |
| *Time between birth and hysterectomy* | ≤48hrs | 40 | (93.0) | 34 | (75.6) | 0.025 |
|  | >48hrs | 3 | (7.0) | 11 | (24.4) |  |
| Conservative approach: placenta left in situ | Yes | 18 | (27.3) | 53 | (56.4) | <0.001 |
|  | No | 48 | (72.7) | 41 | (43.6) |  |
| *How much left in situ* | Complete | 14 | (77.8) | 39 | (73.6) | 0.724 |
|  | Partial | 4 | (22.2) | 14 | (26.4) |  |
| *Hysterectomy after left in situ* | *Yes* | 4 | (22.2) | 17 | (32.1) | 0.429 |
| *Time between birth and hysterectom*y | <48hrs | 1 | (25.0) | 6 | (35.3) | 0.999 |
|  | >48hrs | 3 | (75.0) | 11 | (64.7) |  |
| *Methotrexate used* | *Yes* | 5 | (27.8) | 0 | (0) | 0.001 |
| Pelvic arterial embolisation | Used | 30 | (45.5) | 33 | (35.1) | 0.187 |
| Other conservative surgery* | Used | 13 | (19.7) | 9 | (9.6) | 0.067 |
| Uterine balloon tamponade | Used | 10 | (15.2) | 6 | (6.4) | 0.069 |
| **Haematological management** |  |  |  |  |  |  |
| Whole blood or Red blood cells received n(%) | | 45 | (68.2) | 47 | (50.0) | 0.022 |
| *In women who received whole blood or Red blood cells* | median (IQR) unit | 7 | (4-10) | 5 | (3-10) | 0.309 |
| FFP received n(%) |  | 33 | (50.0) | 33 | (35.1) | 0.060 |
| *In women who received FFP* | median (IQR) unit | 4 | (3-6) | 5 | (2-8) | 0.342 |
| Platelets received n(%) |  | 19 | (28.8) | 10 | (10.6) | 0.003 |
| *In women who received platelets* | median (IQR) unit | 2 | (1-4) | 2 | (1-4) | 0.864 |
| Fibrinogen received e.g cryoprecipitate or fibrinogen conc. | Yes | 15 | (22.7) | 27 | (28.7) | 0.396 |
| Recombinant Factor VIIa used | Yes | 1 | (1.5) | 1 | (1.1) | 0.999 |

*Includes: arterial ligation and uterine compression sutures. Descriptive statistics calculated excluding the missing.

Table S3. Maternal and infant outcomes of women with PAS suspected in the antenatal period.

|  |  | Abnormally invasive placenta suspected in antenatal period | | | | |
| --- | --- | --- | --- | --- | --- | --- |
| **Maternal outcomes** |  | UK n(%) n=66 | | France n(%) n=94 | | P-value |
| Amount of blood loss (mL) | Median (IQR) | 3000 | (1000-6500) | 925 | (500-2000) | <0.001 |
| *Severe postpartum haemorrhage (mL)* | <3000 | 32 | (48.5) | 74 | (82.2) | <0.001 |
|  | ≥3000 | 34 | (51.5) | 16 | (17.8) |  |
|  | *Missing* | 0 |  | 4 |  |  |
| *Major postpartum haemorrhage (mL)* | <2000 | 25 | (37.9) | 66 | (73.3) | <0.001 |
|  | ≥2000 | 41 | (62.1) | 24 | (26.7) |  |
|  | Missing | 0 |  | 4 |  |  |
| Massive Transfusion (units) | ≥6 | 30 | (66.7) | 21 | (45.7) | 0.043 |
|  | <6 | 15 | (33.3) | 25 | (54.3) |  |
|  | *Missing* | 0 |  | 1 |  |  |
| Postpartum infection | Yes | 0 | (0.0) | 2 | (2.1) | 0.233 |
| Damage to bowel, urinary tract and bladder | Yes | 8 | (12.1) | 11 | (11.7) | 0.936 |
|  | *Missing* | 0 |  | 0 |  |  |
| ITU admission | Yes | 53 | (80.3) | 37 | (39.4) | <0.001 |
|  | *Missing* | 0 |  | 0 |  |  |
| Maternal mortality | Yes | 0 | (0.0) | 1 | (1.1) | 0.999 |
| **Infant outcomes** |  | UK n (%) n=66 | | France n (%) n=95 | |  |
| Perinatal mortality | *No* | 65 | (98.5) | 94 | (98.9) | 0.999 |
|  | *Yes* | 1 | (1.5) | 1 | (1.1) |  |
|  | *Missing* | 0 |  | 0 |  |  |

Descriptive statistics calculated excluding the missing.

Table S4. Management and maternal and infant outcomes of women with placenta percreta.

| **Management** |  | UK n(%) n=39 | | France n(%) n=47 | | P-Value |
| --- | --- | --- | --- | --- | --- | --- |
| PAS suspected prior to delivery | Yes | 23 | (59) | 42 | (89.4) | 0.001 |
|  | No | 16 | (41) | 5 | (10.6) |  |
| Caesarean section | Yes | 36 | (92.3) | 47 | (100) | 0.089 |
| Caesarean Hysterectomy | Yes | 19 | (48.7) | 18 | (38.3) | 0.331 |
|  | No | 20 | (51.3) | 29 | (61.7) |  |
| Total Hysterectomy | Yes | 24 | (61.5) | 30 | (63.8) | 0.827 |
|  | No | 15 | (38.5) | 17 | (36.2) |  |
| *Time between delivery and total hysterectomy* | ≤48hrs | 21 | (87.5) | 23 | (76.7) | 0.309 |
|  | >48hrs | 3 | (12.5) | 7 | (23.3) |  |
| No attempt to remove placenta after birth | Attempt | 19 | (48.7) | 8 | (17.4) | 0.002 |
|  | No attempt | 20 | (51.3) | 38 | (82.6) |  |
|  | Missing | 0 |  | 1 |  |  |
| Conservative approach: placenta left in situ | Yes | 13 | (33.3) | 29 | (61.7) | 0.009 |
|  | No | 26 | (66.7) | 18 | (38.3) |  |
|  | Unknown | 0 |  | 0 |  |  |
| *How much left in situ* | Complete | 11 | (84.6) | 23 | (79.3) | 0.686 |
|  | Partial | 2 | (15.4) | 6 | (20.7) |  |
| *Had hysterectomy after left in situ* | *Yes* | 4 | (30.8) | 12 | (41.4) | 0.513 |
| Time between birth and hysterectomy | ≤48hrs | 1 | (25.0) | 5 | (41.7) | 0.999 |
|  | >48hrs | 3 | (75.0) | 7 | (58.3) |  |
| *Methotrexate used* | *Yes* | 5 | (38.5) | 0 | (0) | 0.002 |
| Pelvic arterial embolisation | Not used | 25 | (64.1) | 27 | (57.4) | 0.530 |
|  | Used | 14 | (35.9) | 20 | (42.6) |  |
| Other conservative surgery* | Not used | 28 | (71.8) | 42 | (89.4) | 0.037 |
|  | Used | 11 | (28.2) | 5 | (10.6) |  |
| Whole blood or Red blood cells received n(%) |  | 28 | (71.8) | 30 | (63.8) | 0.433 |
| *In women who received whole blood or Red blood cells* | median (IQR) unit | 8 | (6-14) | 6 | (3-11) | 0.203 |
| FFP received n(%) |  | 20 | (51.3) | 26 | (55.3) | 0.709 |
| *In women who received FFP* | median (IQR) units | 4 | (4-8) | 4 | (2-8) | 0.383 |
| Platelets received n(%) |  | 13 | (33.3) | 7 | (14.9) | 0.044 |
| *In women who received platelets* | median (IQR) units | 2 | (1-2) | 2 | (1-8) | 0.382 |
| Fibrinogen received e.g cryoprecipitate or fibrinogen conc. | Not used | 29 | (74.4) | 30 | (63.8) | 0.295 |
|  | Used | 10 | (25.6) | 17 | (36.2) |  |
| Maternal mortality | Yes | 0 | (0) | 1 | (2.1) | 0.999 |
| Amount of blood loss (mL) | Median (IQR) | 3000 | (1500-9000) | 1200 | (500-3100) | <0.001 |
| *Severe postpartum haemorrhage* | <3000 | 18 | (46.2) | 32 | (71.1) | 0.020 |
|  | ≥3000 | 21 | (53.8) | 13 | (28.9) |  |
|  | *Missing* | 0 |  | 2 |  |  |
| *Major postpartum haemorrhage* | <2000 | 12 | (30.8) | 29 | (64.4) | 0.002 |
|  | ≥2000 | 27 | (69.2) | 16 | (35.6) |  |
|  | Missing | 0 |  | 2 |  |  |
| Massive transfusion | ≥ 6 | 21 | (75.0) | 16 | (55.2) | 0.117 |
|  | < 6 | 7 | (25.0) | 13 | (44.8) |  |
|  | Missing | 0 |  | 1 |  |  |
| Postpartum infection | Yes | 0 | (0.0) | 7 | (14.9) | 0.015 |
| Damage to bowel, urinary tract and bladder | Yes | 6 | (15.4) | 8 | (17) | 0.838 |
| ITU admission | Yes | 31 | (79.5) | 19 | (40.4) | <0.001 |
| **Infant outcomes** |  | UK n(%) n=39 | | France n(%) n=47 | |  |
| Perinatal mortality | *Yes* | 1 | (2.8) | 1 | (2.13) | 0.999 |
|  | *Missing* | 3 |  | 0 |  |  |

* Includes: arterial ligation and uterine compression sutures. Descriptive statistics calculated excluding the missing.

*Table S5. Management and maternal and infant outcomes of women who had antenatally detected placenta praevia.*

| **Management** |  | UK n(%) n=86 | | France n(%) n=138 | | P-Value |
| --- | --- | --- | --- | --- | --- | --- |
| PAS suspected prior to birth | Yes | 64 | (74.4) | 86 | (65.2) | 0.149 |
|  | No | 22 | (25.6) | 46 | (34.8) |  |
|  | Missing | 0 |  | 6 |  |  |
| Caesarean section | Yes | 83 | (96.5) | 132 | (95.7) | 0.750 |
| Caesarean hysterectomy | Yes | 46 | (53.5) | 46 | (33.3) | 0.003 |
|  | No | 40 | (46.5) | 92 | (66.7) |  |
|  | Missing | 0 |  | 0 |  |  |
| Total hysterectomy | Yes | 56 | (65.1) | 66 | (48.2) | 0.013 |
|  | No | 30 | (34.9) | 71 | (51.8) |  |
|  | Missing | 0 |  | 1 |  |  |
| *Time between birth and hysterectomy* | ≤48hrs | 53 | (94.6) | 53 | (80.3) | 0.019 |
|  | >48hrs | 3 | (5.4) | 13 | (19.7) |  |
| No attempt to remove placenta after birth | Attempt | 58 | (67.4) | 62 | (45.3) | 0.001 |
|  | No attempt | 28 | (32.6) | 75 | (54.7) |  |
|  | Missing | 0 |  | 1 |  |  |
| Conservative approach: placenta left in situ | Yes | 20 | (23.3) | 59 | (42.8) | 0.003 |
|  | No | 66 | (76.7) | 79 | (57.2) |  |
|  | Unknown |  |  | 2 |  |  |
| *How much left in situ* | Complete | 15 | (75.0) | 36 | (61) | 0.259 |
|  | Partial | 5 | (25.0) | 23 | (39) |  |
| *Had hysterectomy after left in situ* | Yes | 5 | (25.0) | 18 | (30.5) | 0.639 |
| Pelvic arterial embolisation | Used | 30 | (34.9) | 39 | (28.3) | 0.296 |
| Other conservative surgery* | Used | 18 | (20.9) | 23 | (16.7) | 0.422 |
| Whole blood or Red blood cells received n(%) | | 64 | (74.4) | 77 | (55.8) | 0.005 |
| *In women who received whole blood or Red blood cells* | median (IQR) unit | 7 | (4-12) | 6 | (3-11) | 0.373 |
| FFP received n(%) |  | 47 | (54.7) | 60 | (43.5) | 0.103 |
| *In women who received FFP* | median (IQR) units | 4 | (4-8) | 5 | (2-8) |  |
| Platelets received n(%) |  | 29 | (33.7) | 20 | (14.5) | 0.001 |
| *In women who received platelets* | median (IQR) units | 2 | (1-2) | 1 | (1-2) |  |
| Fibrinogen received | Used | 21 | (24.4) | 48 | (34.8) | 0.102 |
| **Maternal outcomes** |  |  |  |  |  |  |
| Maternal mortality | Yes | 0 | (0.0) | 1 | (0.7) | 0.999 |
| Amount of blood loss *(mL)* | Median (IQR) | 3000 | (1500-7000) | 1200 | (500-2500) | |
| *Severe postpartum haemorrhage (mL)* | <3000 | 38 | (44.2) | 100 | (76.3) | <0.001 |
|  | ≥3000 | 48 | (55.8) | 31 | (23.7) |  |
|  | *Missing* | 0 |  | 7 |  |  |
| *Major postpartum haemorrhage (mL)* | <2000 | 25 | (29.1) | 85 | (64.9) | <0.001 |
|  | ≥2000 | 61 | (70.9) | 46 | (35.1) |  |
|  | Missing | 0 |  | 7 |  |  |
| Massive transfusion (units) | ≥6 | 43 | (67.2) | 38 | (50.7) | 0.049 |
|  | < 6 | 21 | (32.8) | 37 | (49.3) |  |
|  | Missing | 0 |  | 2 |  |  |
| Postpartum infection | Yes | 2 | (2.3) | 3 | (2.2) | 0.953 |
| Damage to bowel, urinary tract and bladder | Yes | 10 | (11.6) | 15 | (11) | 0.891 |
| ITU admission | Yes | 67 | (77.9) | 50 | (36.5) | <0.001 |
| **Infant outcomes** |  | UK n(%) n=86 | | France n(%) n=138 | |  |
| Perinatal mortality | *Yes* | 2 | (2.3) | 3 | (2.2) | 0.999 |
|  | *Missing* | 1 |  | 3 |  |  |

* Includes: arterial ligation and uterine compression sutures. Descriptive statistics calculated excluding the missing.

Table S6. Pregnancy characteristics and haematological management of women with PAS

| **Pregnancy characteristics** |  | **UK n(%) n=134** |  | **France n(%) n=219** |  | P-value |
| --- | --- | --- | --- | --- | --- | --- |
| Termination of pregnancy | Yes | 2 | (1.5) | 1 | (0.5) | 0.559 |
|  | *Missing* | 2 |  | 0 |  |  |
| Gestational age at birth | <34 weeks | 23 | (17.2) | 39 | (17.8) | 0.874 |
|  | ≥34 & <37 weeks | 43 | (32.1) | 75 | (34.2) |  |
|  | ≥37 weeks | 68 | (50.7) | 105 | (47.9) |  |
| Planned caesarean section | Yes | 110 | (83.3) | 138 | (63.0) | <0.001 |
|  | *Missing* | 2 |  | 0 |  |  |
| **Haematological management** |  |  |  |  |  |  |
| FFP received n (%) |  | 69 | (51.5) | 88 | (40.2) | 0.038 |
| *In women who received FFP* | median (IQR) units | 4 | (4-7) | 4 | (2-8) | 0.716 |
| Platelets received n (%) |  | 43 | (32.1) | 29 | (13.2) | <0.001 |
| *In women who received platelets* | median (IQR) units | 2 | (1-2) | 1 | (1-4) | 0.859 |
| Fibrinogen received e.g. cryoprecipitate or fibrinogen conc. | Yes | 34 | (25.4) | 69 | (31.5) | 0.219 |
| Recombinant Factor VIIa used | Yes | 5 | (3.7) | 3 | (1.4) | 0.148 |

Descriptive statistics calculated excluding the missing.
