# Supplementary material for: Placenta accreta spectrum – variations in clinical practice and maternal morbidity between the UK and France: a population‐based comparative study
Source: BJOG. 2022 Apr 29;129(10):1676–85. doi: 10.1111/1471-0528.17169 (PMC9544707; doi:10.1111/1471-0528.17169)
Supplement: Supplementary file 2 — Appendix S1 [file BJO-129-1676-s004.docx]

# Supplementary methods section

##### Case selection

A harmonised case definition was selected using the cases from the PACCRETA study that met the stricter UKOSS definition (Box 1). The UKOSS definition did not include women who had a haemorrhage as a result of a manual removal of placenta; therefore, there were 16 women from notified UKOSS cases that did not meet the case definition (27). In those who only met the fourth criteria of the PACCRETA study (antenatal diagnosis and at laparotomy), their PACCRETA forms were checked to assess if they met the UKOSS definition. These cases were included if they had a placenta percreta or surgical management for haemorrhage which included a hysterectomy and conservative surgical management.

##### Comparability of datasets

The PACCRETA data collection form was translated into English, and all data items in the UKOSS data collection form were mapped to the PACCRETA data collection form. The characteristics, management and outcomes of interest were identified from the literature, and if these were available and comparable between datasets, they were extracted. For variables where there was not a uniform definition, a common definition was generated. If this was not possible, then the variable was excluded from the analysis.
